# Granulomatous Lung Inflammation of Unknown Causes Following COVID-19

**DOI:** 10.7759/cureus.107322

**Published:** 2026-04-19

**Authors:** Keisuke Watanabe, Takeshi Kaneko

**Affiliations:** 1 Department of Pulmonary Medicine, International University of Health and Welfare, Atami Hospital, Atami, JPN; 2 Department of Pulmonology, Graduate School of Medicine, Yokohama City University, Yokohama, JPN

**Keywords:** coronavirus disease 2019, granuloma, non caseating granuloma, post-covid-19, sarcoidosis

## Abstract

Introduction

Several studies have reported that COVID-19 increases the risk of autoimmune diseases, including sarcoidosis. Recently, we reported cases of pulmonary granulomatous inflammation distinct from sarcoidosis following COVID-19.Based on these observations, we hypothesize that COVID-19 may induce pulmonary granulomatous inflammation distinct from sarcoidosis.

Methods

We conducted a retrospective review of the medical records of adult patients (≥18 years) with pathologically confirmed noncaseating granulomas in the lung and/or thoracic lymph nodes (hilar or mediastinal) between January 2020 and August 2023.

Results

Fifty-four cases with pathologically confirmed noncaseating granulomas were identified. Specimens showing noncaseating granulomas were obtained by bronchoscopy in 36 cases (66.7%) and by surgery in 18 cases (33.3%). Three cases (5.6%) of noncaseating granulomas of unknown etiology were identified. Two of the three cases (3.7%) had a history of COVID-19 prior to the development of pulmonary granulomatous inflammation.

Conclusion

In the study, 5.6% of cases of noncaseating granulomatous inflammation in the lung and/or thoracic lymph nodes were of unknown etiology, and two of these cases (3.7%) had antecedent COVID-19. These findings suggest that COVID-19 may be associated with the development of pulmonary granulomatous inflammation distinct from sarcoidosis.

## Introduction

Several studies have reported that COVID-19 increases the risk of autoimmune diseases, including sarcoidosis [1,2]. Sarcoidosis is a systemic disease characterized by granuloma formation; however, granulomas can also arise in conditions other than sarcoidosis, such as those associated with infections, drugs, and malignancies [3]. Previous studies have reported that certain viral infections can lead to post-infectious granulomatous inflammation [4,5]. Recently, we reported cases of pulmonary granulomatous inflammation distinct from sarcoidosis following COVID-19 [6]. Based on these observations, we hypothesize that COVID-19 may induce pulmonary granulomatous inflammation distinct from sarcoidosis. Therefore, we retrospectively analyzed the subjects with pathologically confirmed noncaseating granulomas at our hospital. This study aimed to determine the proportion and clinical characteristics of noncaseating granulomas of unknown etiology and to evaluate their association with prior COVID-19.

## Materials and methods

Study population

This study was undertaken at Yokohama City University Hospital. We conducted a retrospective review of the medical records of adult patients (≥18 years) with pathologically confirmed noncaseating granulomas in the lung and/or thoracic lymph nodes (hilar or mediastinal) between January 2020 and August 2023. The start date of January 2020 was selected because the first case of COVID-19 in Japan was reported on January 15, 2020. Clinical, laboratory, radiological, and pathological data were obtained from the electronic medical records, including age, sex, smoking history, medical history, occupation, and medication use. 

Diagnostic methods

Biopsy specimens were obtained by surgical biopsy or bronchoscopy (transbronchial lung biopsy and/or endobronchial ultrasound-guided transbronchial needle aspiration). Pathological findings were diagnosed by the board-certified pathologists at our hospital. Chest CT was obtained in all the subjects. Radiological findings were evaluated by the board-certified radiologists at our hospital.

Sarcoidosis was diagnosed according to the joint statement of the American Thoracic Society, the European Respiratory Society, and the World Association of Sarcoidosis and Other Granulomatous Disorders [7]. Non-tuberculous mycobacterial lung disease was diagnosed based on the criteria of the American Thoracic Society and the Infectious Diseases Society of America [8]. Interstitial pneumonia was diagnosed based on radiological findings, with or without pathological confirmation. Sarcoid reaction was diagnosed based on localized granulomatous lesions and the presence of conditions known to induce sarcoid reactions (e.g., malignancy, infectious diseases, and drugs). Fungal infection was diagnosed based on serological tests and/or the detection of pathogens in respiratory samples. Non-caseating granulomas of unknown etiology were diagnosed based on clinical, laboratory, radiological, and pathological findings that indicated no specific cause. In cases of diagnostic uncertainty, the diagnosis was established by multidisciplinary discussion involving pulmonologists, pathologists, and radiologists at our hospital.

COVID-19 was confirmed by a polymerase chain reaction (PCR) test or an antigen test using a nasal swab. The interval from COVID-19 diagnosis to the appearance of pulmonary opacity was defined as the time from confirmation of COVID-19 to the detection of opacity.

Statistical analysis

Statistical analyses were performed using EZR (spelled Easy R, Jichi Medical University, Tochigi, Japan) [9]. Categorical variables were compared using Fisher’s exact test. A P value <0.05 was considered statistically significant, and all tests were two-tailed.

Ethical statement

This study was approved by the institutional review board of our institution (approval No. F231100006). Owing to the retrospective design of the study, the requirement for written informed consent was waived.

## Results

Fifty-four cases with pathologically confirmed noncaseating granulomas were identified. Specimens showing noncaseating granulomas were obtained by bronchoscopy in 36 cases (66.7%) and by surgery in 18 cases (33.3%) (Table 1).

**Table 1:** Baseline demographic and procedural characteristics (n=54) Data are presented as median (range) or number of the patients (%), if not otherwise specified. ^a^Thoracic lymph nodes include hilar or mediastinal lymph nodes. ^b^Bronchoscopy includes transbronchial lung cryobiopsy, transbronchial biopsy and ultrasound transbronchial needle aspirations.

Variable	N
Female:Male	23:31
Age (years)	67.5 (39-83)
Smoking history	
Current	19 (35.2%)
Previous	30 (55.6%)
Never	5 (9.3%)
Biopsy site	
Lung	24 (44.4%)
Lung and thoracic lymph nodes^a^	6 (11.1%)
Thoracic lymph nodes	24 (44.4%)
Biopsy procedure	
Bronchoscopy^b^	36 (66.7%)
Surgery	18 (33.3%)

**Figure 1:**
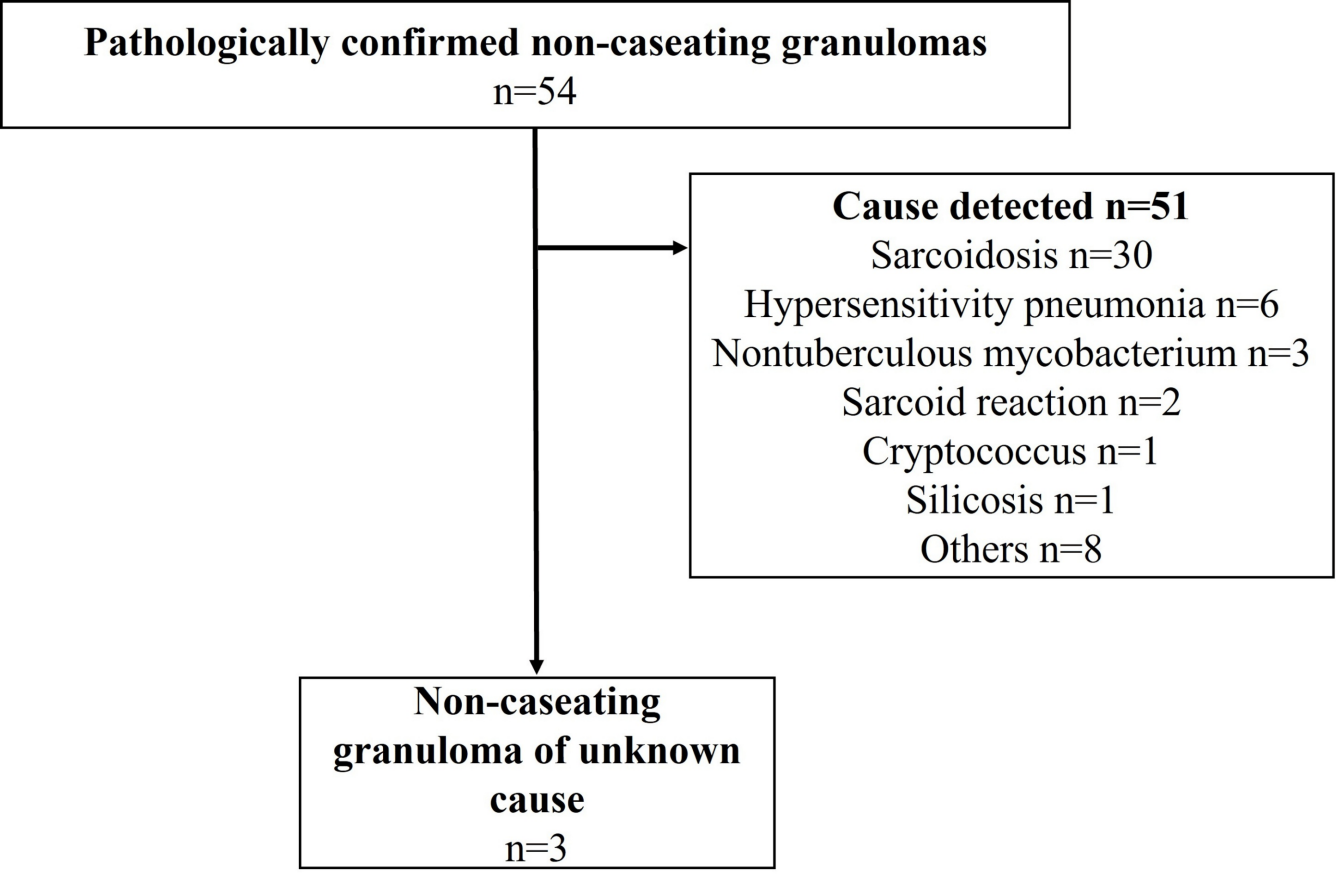
Flow diagram of the study “Others” included other types of interstitial pneumonia (n=7), defined as secondary interstitial pneumonia excluding hypersensitivity pneumonia, cryptogenic organizing pneumonia, and unclassifiable idiopathic interstitial pneumonia, as well as undifferentiated connective tissue disease (n=1).

Biopsy sites included the lung in 24 cases (44.4%), both the lung and thoracic lymph nodes in six cases (11.1%), and thoracic lymph nodes alone in 24 cases (44.4%). Of these, 51 cases were excluded because an underlying causative disease was identified (Figure 1).

Consequently, three cases (5.6%, 95% confidence interval (CI): 1.2-15.4%) of noncaseating granulomas of unknown etiology were identified. No significant difference was observed in the proportion of granulomas of unknown etiology among biopsy sites (lung vs. thoracic lymph nodes vs. lung and thoracic lymph nodes, 12.5% vs. 0% vs. 0%, P=0.327). There was no significant difference in the proportion of granulomas of unknown etiology between cases diagnosed by bronchoscopy and those diagnosed by surgical biopsy (bronchoscopy vs. surgical biopsy, 2.8% vs. 11.1%, P=0.255) (Table 2).

**Table 2:** The proportion of granulomas of unknown etiology by the biopsy site and procedure. ^a, b^Fisher’s exact test was used.

Variable	The proportion of granulomas of unknown etiology (%)	P
Biopsy site		0.327^a^
Lung	3/24 (12.5%)	
Lung and thoracic lymph nodes	0/6 (0%)	
Thoracic lymph nodes	0/24 (0%)	
Biopsy procedure		0.255^b^
Bronchoscopy	1/36 (2.8%)	
Surgery	2/18 (11.1%)	

Details of three cases are shown in Table 3.

**Table 3:** Characteristics of non-caseating granuloma of unknown cause SLB: Surgical lung biopsy; TBB: Transbronchial biopsy.

Case	1	2	3
Age	69	50	55
Sex	Male	Male	Male
Smoking history	Past	Past	Past
Dust exposure	No	No	No
Cancer	No	No	No
COVID-19 (COVID-19 to appearance of opacity)	Yes (3 months)	No	Yes (3 months)
Radiological findings	Mass-like	Mass-like	Multiple nodules
Biopsy site	Lung	Lung	Lung
Biopsy procedure	TBB	SLB	SLB
Prognosis	Spontaneous remission	Spontaneous remission	Spontaneous remission

Two of the three cases (3.7%, 95% CI: 0.5-12.7%) had a history of COVID-19 prior to the development of pulmonary granulomatous inflammation.

## Discussion

In this study, 5.6% of cases of noncaseating granulomatous inflammation of the lung were of unknown etiology, and two of the three subjects with noncaseating granulomas of unknown etiology had a history of COVID-19. The reported proportion of pulmonary granulomas of unknown etiology ranges from 3.8% to 42%, with substantial variability across studies [10-13]. Moreover, the underlying causes differ markedly by region [10]. Given the small number of cases in the present study, meaningful comparisons are limited. Whether the distribution of underlying causes of pulmonary granulomas has changed, or whether the proportion of granulomas of unknown etiology has increased since the onset of the COVID-19 pandemic, warrants further investigation.

We suspected that COVID-19 caused post-infectious pulmonary granulomatous inflammation in these two cases. No bacteria or fungi were detected on bronchial wash cultures (Case 1) or tissue cultures (Case 3). Blood screening for fungal infections, including β-D-glucan, *Aspergillus* antigen, and *Cryptococcus* antigen, was negative in both cases. Other potential underlying diseases were also excluded based on clinical and laboratory findings. Although several studies have suggested that sarcoidosis may develop after COVID-19 [1,2], the clinical and pathological features of these subjects were atypical for sarcoidosis. Case 1 presented with a solitary peripheral consolidation in the right upper lobe, whereas a solitary lung mass is an unusual radiologic manifestation of sarcoidosis [14]. Case 3 showed multiple bilateral pulmonary nodules without mediastinal or hilar lymphadenopathy. Nodular sarcoidosis is an uncommon subtype, accounting for only 2.4%-4% of sarcoidosis cases [15]. Moreover, Malaisamy et al. reported that mediastinal and hilar lymphadenopathy is observed in most cases of nodular sarcoidosis [15]. The pathological findings were also atypical for sarcoidosis. Histopathological features not suggestive of sarcoidosis include loosely organized collections of mononuclear phagocytes or multinucleated giant cells, extensive necrosis, and a lack of lymphatic distribution of granulomas [16]. In the present study, pathological specimens showed peribronchiolar noncaseating granulomas in Case 1 and loosely formed noncaseating granulomas in the alveolar spaces in Case 3, both of which are inconsistent with typical sarcoidosis. Detailed descriptions of these two cases have been reported elsewhere [6].

Vij et al. reported a review of sarcoidosis following COVID-19, in which most patients were treated with systemic corticosteroids [17]. In contrast, the two cases in our study with noncaseating granulomas of unknown etiology following COVID-19 recovered spontaneously without systemic therapy. This difference in clinical course may further suggest a distinction between sarcoidosis following COVID-19 and noncaseating granulomatous inflammation of unknown etiology occurring after COVID-19.

This study has several limitations. First, it was a retrospective, single-center study with a small sample size. Second, the severe acute respiratory syndrome coronavirus 2 (SARS-CoV-2) variants could not be identified, although the clinical features of COVID-19 are known to vary among variants. Third, a diagnosis of sarcoidosis could not be completely excluded, as atypical presentations of sarcoidosis have been reported [14]. Fourth, it was difficult to determine how many subjects with granulomas of known etiology had a prior history of COVID-19. In addition, pre-COVID-19 chest imaging and clinical data were unavailable for the two cases, and thus the presence of pulmonary granulomas before COVID-19 could not be ruled out. Fifth, because this was a retrospective study, no predefined protocol was used to determine the cause of noncaseating granulomas. However, all diagnoses were reviewed by the authors.

## Conclusions

In conclusion, 5.6% of cases of noncaseating granulomatous inflammation in the lung and/or thoracic lymph nodes were of unknown etiology, and two of these cases (3.7%) had antecedent COVID-19. These findings suggest that COVID-19 may be associated with the development of pulmonary granulomatous inflammation distinct from sarcoidosis. Further studies are warranted to clarify whether COVID-19 can cause noncaseating pulmonary granulomas.
